# Propofol Exposure Precipitating a Functional Movement Disorder: A Case Report

**DOI:** 10.1155/cria/8850185

**Published:** 2025-08-16

**Authors:** Shahbaz Saad, Federico Ciardi, Nathan Praschan, Brittani Bungart, Michael Fettiplace

**Affiliations:** ^1^ Department of Anesthesiology, University of Texas Health Science Center, Houston, Texas, USA, uth.edu; ^2^ Department of Anesthesiology, Weill Cornell Medicine, New York, New York, USA, cornell.edu; ^3^ Department of Psychiatry, Division of Neuropsychiatry, Brigham and Women’s Hospital, Boston, Massachusetts, USA, brighamandwomens.org; ^4^ Department of Anesthesiology, Vanderbilt University Medical Center, Nashville, Tennessee, USA, vanderbilt.edu; ^5^ Department of Anesthesiology, University of Illinois at Chicago, Chicago, Illinois, USA, uic.edu

**Keywords:** acute dystonic reaction, functional neurological disorder, involuntary movements, movement disorder, parkinsonism

## Abstract

Drug‐induced movement disorders are a rare but distressing complication of many anesthetic agents. When such reactions occur, it is difficult to identify a causative agent. We report a case of recurrent ataxia, dysarthria, chorea, and tremors following multiple anesthetics in a 32‐year‐old patient with a history of significant postoperative nausea and vomiting. Over the course of four anesthetics, the exclusion of propofol, along with collaborative planning via a biopsychosocial approach, prevented the onset of a functional movement disorder. Therefore, for the first time, we highlight a case of functional movement disorder precipitated by intraoperative propofol.

## 1. Introduction

Modern anesthesia uses multiple drugs to achieve amnesia, akinesia, analgesia, and hypnosis. Identifying the causative agent for an adverse drug reaction is often challenging, particularly when using multiple simultaneous medications perioperatively. Since the introduction of propofol in the 1980s, practitioners have reported cases of involuntary movements associated with propofol [1]. Despite this, propofol is sometimes overlooked as a causative agent postoperatively, especially since it is used as a therapy for treatment‐resistant epilepsy and some movement disorders. We report a case of a recurrent anesthesia‐associated movement disorder successfully treated by avoidance of propofol and collaborative planning via a biopsychosocial approach. Health Insurance Portability and Accountability Act (HIPAA) authorization was obtained from the patient to publish this case report, and we adhere to the CARE statement.

## 2. Case Report

A 32‐year‐old, 183 cm, 79 kg, Caucasian woman with a history of supraventricular tachycardia, temporomandibular joint (TMJ) arthritis, fibromyalgia, anxiety, migraines, postoperative nausea and vomiting (PONV), and postanesthesia movement disorders presented for TMJ arthroscopy and follow‐up TMJ replacement. There was no personal or family history of any seizure disorder. Home medications included methotrexate 20 mg weekly, colchicine 0.6 mg daily, pregabalin 50 mg nightly, and folic acid 1000 mg daily for fibromyalgia; duloxetine 20 mg daily and lorazepam 0.5 mg as needed for anxiety; 2.5 mg of zolmitriptan for migraines; over‐the‐counter famotidine 20 mg BID for reflux; medroxyprogesterone depot injection 150 mg every 12 weeks for birth control; and 25 mg diphenhydramine on the day of surgery for additional anxiety. She denied tobacco, alcohol, or recreational drug use.

On presentation for TMJ arthroscopy in September of 2020, the patient reported multiple anesthetics with abnormal movement occurring after emergence and requested collaborative planning to avoid another event. The first episode occurred following a catheter ablation for supraventricular tachycardia 12 years prior. Two days postoperatively, she developed headache, nausea, vomiting, and retro‐orbital eye pain without visual disturbances, potentially representing an ocular migraine or trigeminal neuralgia. In the emergency room a noncontrast head computed tomography scan and lumbar puncture yielded no abnormalities. She was diagnosed with a migraine and treated symptomatically for nausea with 4 mg of intravenous ondansetron and treated for pain with 4 mg of intravenous morphine. Subsequently, she developed facial droop, dysarthria, ataxia, and whole‐body tremors (Supporting Video 1). She was admitted and diagnosed with conversion disorder (now reclassified as a functional neurological disorder [FND]). Two weeks later, an independent neurologist evaluated the patient and diagnosed her with drug‐induced parkinsonism due to ondansetron. Intravenous diphenhydramine rapidly alleviated her symptoms. The second episode occurred following regional anesthesia with propofol sedation for an ankle arthroscopy in January 2020. She noticed tremors on emergence for which she received diphenhydramine, clonidine, and diazepam (Table 1). The movements stopped, and she was discharged, but symptoms recurred the following morning. She was admitted and retreated with intravenous diphenhydramine, which terminated the tremors. A neurology consultation diagnosed a FND, citing variable frequency, amplitude, and presence of distractibility and entrainment. The movements recurred and persisted for a week with spontaneous resolution.

**Table 1:** Perioperative drug exposures during eight anesthetics between 2008 and 2022, and the incidence of postoperative involuntary movements.

Operation [date]	SVT focus ablation [1/2008]	Ankle arthroscopy [1/2020]	TMJ arthroscopy [9/2020]	TMJ arthroscopy [10/2021]	Maxillary artery embolization [7/2022]	TMJ arthroplasty [7/2022]
*Intraoperative drug*						
Acetaminophen		Y	Y			Y
Bupivacaine		Y				
Cefazolin				Y		Y
Clindamycin		Y	Y			
Clonidine		Y				
Cyclobenzaprine			Y			
Dexamethasone		Y	Y		Y	Y
Dexmedetomidine				Y	Y	
Diazepam		Y	Y			
^#^Diphenhydramine	Y	Y	Y			
Fentanyl	Y	Y			Y	Y
Gabapentin		Y		Y		
Glycopyrrolate	Y					
Haloperidol						Y
Hydromorphone				Y		
Ketamine		Y				Y
Ketorolac		Y				Y
Lidocaine	Y	Y	Y		Y	Y
Meperidine			Y			
Methadone						Y
Midazolam		Y	Y	Y	Y	Y
Morphine	Y					Y
Neostigmine	Y					
Prochlorperazine				Y		
^*#*^ *Propofol*	*Y*	*Y*	*Y*			
Ondansetron	Y				Y	Y
Oxycodone				Y		
Remifentanil			Y			Y
Rocuronium			Y	Y	Y	
Sevoflurane	Y			Y	Y	Y
Scopolamine		Y	Y			Y
Succinylcholine						Y
Sugammadex				Y	Y	
Vecuronium	Y					
**Involuntary movements**	Y	Y	Y			

*Note:* SVT = supraventricular tachycardia. TMJ = temporomandibular joint. Y: drug used during the case or involuntary movements present.

^#^Agents associated with abnormal movements but not lack of abnormal movements.

On presentation for her initial TMJ arthroscopy, we planned for the possibility of a drug‐induced movement disorder versus a FND precipitated by anesthesia exposure. We premedicated with multiple agents used for movement disorders (diphenhydramine, diazepam, and cyclobenzaprine), omitted ondansetron as a potential trigger, and avoided dissociative drugs like ketamine that might precipitate a functional event [2]. A total intravenous anesthetic with propofol and remifentanil was employed, given the patient’s history of PONV (Table 1) along with dexamethasone, diphenhydramine, and a scopolamine patch. Immediately after emergence, she developed violent tremors and dystonia that persisted throughout the hospitalization (Supporting Video 1). A neurology consultation re‐diagnosed a FND citing variable frequency, amplitude, and presence of distractibility and entrainment. The painful movements persisted for weeks and eventually resolved spontaneously. For all cases, she was normothermic and normocarbic, without evidence of infection, rigidity, hyper‐reflexia, or autonomic instability.

She ultimately required a second TMJ arthroscopy, for which the patient was very anxious and re‐requested collaborative planning. She provided videos of prior episodes that demonstrated movements consistent with an FND (Supporting Video 1). Given the profound reaction following her total intravenous anesthetic with a large propofol dose, we considered propofol as a precipitating agent [1, 3, 4] and avoided propofol [5]. The anesthetic proceeded uneventfully with the agents listed in Table 1, along with surgeon‐delivered lidocaine. In the postrecovery anesthesia unit, she experienced profound PONV and received prochlorperazine. She demonstrated no abnormal motor movements and reported that the PONV was preferable to abnormal movements. In July 2022, she followed up for right maxillary artery embolization and TMJ replacement the following day. For both anesthetics she received no propofol and experienced no abnormal movements with medications listed in Table 1, despite the reintroduction of multiple agents potentially associated with drug‐induced movement disorders.

## 3. Discussion

We present a case of postanesthetic functional movement disorder successfully prevented with propofol avoidance. While general anesthesia is associated with FNDs [2, 6], and propofol causes abnormal movements [1, 3], propofol has not been identified as a primary causative agent. During six procedures, propofol and diphenhydramine were the only drugs associated with abnormal movements. For these cases, propofol preceded the abnormal movements, whereas diphenhydramine was used as an abortive or preventative agent. Most cases of propofol‐associated movement disorders categorize the movements as either dystonic or epileptiform [3, 4], but without a causative pathophysiology. In some reports, there are alternative diagnoses with concrete pathophysiology, including local anesthetic systemic toxicity [7] or serotonin syndrome [8]. Other cases involve prolonged movement disorders with a morphology similar to our case [9] but without a unifying explanatory etiology. We suggest they may be FNDs as well.

The phenomenology of the patient’s movements—characterized by profound fluctuations in intensity and type, distractibility and entrainment, and hallmark features of functional gait that are diagnostic of FND with abnormal movement—was corroborated by multiple independent neurologists. Singular incidents like trauma, surgery, or exposure to medications may precipitate the onset of FND, consistent with our case [2, 6]. Effective treatment of FND involves a collaborative, multidisciplinary approach that validates the patient’s experience and promotes an understanding of the connection between mind and brain, often alongside physical and occupational therapy [10]. However, anesthetic drugs, including propofol, may precipitate FND events in vulnerable individuals via abreactions or alterations in somatic sensations and emotion processing during emergence [2]. When considering the treatment of propofol‐associated movement disorders, multiple authors suggest diphenhydramine and benztropine, but the sedating effects of these drugs may also provide anxiolysis for FNDs.

We considered a broad differential diagnosis despite the positive features of the movements confirming a diagnosis of FND. Computerized tomography from the initial encounter excluded structural etiologies. The initial lumbar puncture, normothermia throughout all procedures, and lack of leukocytosis excluded infectious etiologies. There was a low likelihood of LAST given the small doses of local anesthetic and lack of other symptoms of LAST. The absence of neuromuscular excitation or autonomic dysfunction argued against serotonin syndrome. Negative family or personal history, normal core body temperatures, normal EtCO_2_, absence of rigidity, and lack of a precipitating agent argued against malignant hyperthermia. Negative social history argued against drug intoxication or withdrawal. The successful reintroduction of multiple drugs also argued against a drug‐induced reaction from ondansetron, haloperidol, prochlorperazine, scopolamine, fentanyl, ketamine, sevoflurane, or any of the other tested drugs. Electroencephalogram at the time of involuntary movements was not obtained, although the movements would be unusual for an epileptic event. Furthermore, multiple independent neurologists evaluated the patient, none of whom recommended an additional workup for epilepsy but instead diagnosed a FND based on bedside evaluation and subsequent review of the supplied videos. The patient was also treated repeatedly with benzodiazepines that could terminate seizures without a profound effect on the symptomology. As such, the case garners 11 points on the Naranjo scale for adverse drug reactions, associated with a definitive effect.

In summary, we present a case of recurrent anesthesia‐associated movement disorder, likely functional in nature. Acknowledgement of our patient’s concerns and shared decision‐making around propofol avoidance may have prevented manifestation of postoperative movements. An integrated biopsychosocial approach may assist with prevention of perioperative FND, but this case highlights that propofol may precipitate FND in vulnerable patients more frequently than other anesthetic agents.

**Nomenclature**
TMJ: Temporomandibular joint
PONV: Postoperative nausea and vomiting
CT: Computed tomography
COMT: Catechol‐O‐methyltransferase
FND: Functional neurological disorder
LAST: Local Anesthetic Systemic Toxicity
EtCO_2_
: End‐tidal concentration of carbon dioxide.

## Consent

This case report was published with the written consent of the patient Dr. Michael Fettiplace.

## Patient Perspective

Thanks for all your help it means more to me than you’ll ever know. Finally having an answer to why this was happening is so relieving and finally hearing that it’s not “all psychological” which I was hearing from neurologist after neurologist is so comforting. Again, THANK YOU!

## Conflicts of Interest

Dr. Brittani Bungart is co‐founder of Bitaic, Inc. and is co‐founder, board member and company officer of Stornamics, Inc. The other authors declare no conflicts of interest.

## Funding

This study was funded by the National Institute of General Medical Sciences, 5T32GM007592‐42.

## Supporting Information

Supporting Digital Content, Video 1: Series of patient‐provided video footage of involuntary movements following multiple anesthetics between 2008 and September 2020. POD = postoperative day.

## Supporting information


**Supporting Information** Additional supporting information can be found online in the Supporting Information section.

## Data Availability

Data are available from the primary authors upon reasonable request.
